# Cushing’s Disease Management: Glimpse Into 2051

**DOI:** 10.3389/fendo.2022.943993

**Published:** 2022-07-06

**Authors:** Rinkoo Dalan, Stefan R. Bornstein, Bernhard O. Boehm

**Affiliations:** ^1^ Tan Tock Seng Hospital, National Healthcare Group, Singapore, Singapore; ^2^ Lee Kong Chian School of Medicine, Nanyang Technological University, Singapore, Singapore; ^3^ Department of Medicine III, University Hospital Carl Gustav Carus, Dresden, Germany; ^4^ Division of Diabetes and Nutritional Sciences, Faculty of Life Sciences and Medicine, King’s College London, London, United Kingdom; ^5^ Klinik für Endokrinologie, Diabetologie und Klinische Ernährung, University Hospital, Zürich, Switzerland

**Keywords:** Cushings disease, circadian rhythms, digital, future, technology development

## Abstract

Major advancements are expected in medicine and healthcare in the 21st century- “Digital Age”, mainly due to the application of data technologies and artificial intelligence into healthcare. In this perspective article we share a short story depicting the future Cushings’ Disease patient and the postulated diagnostic and management approaches. In the discussion, we explain the advances in recent times which makes this future state plausible. We postulate that endocrinology care will be completely reinvented in the Digital Age.

## Short Story: The Midnight Sun

**23 June 2051**

**00:00**:

Sound exposure: limited to the breathing sounds & background noise

Light exposure: no blue light exposure.

Heart Rate: Normal

Systolic Blood Pressure: Trending higher at night

Diastolic Blood Pressure: Trending higher at night

Glucose: Trending higher

Hypnograph: Stage 3/Stage 4 sleep.

Stress level: “High”

Cortisol Level: “High” from 00:00 to 24:00

**23 June 2051**

**06:00**:

The alarm gently rings and plays “Good morning”.

Sunlight creeps through the curtains and fills the room with warmth and light.

Claire awakens, rubbing her eyes seeing the analytics of the previous day affirming good productivity and excellent sleep patterns. A red notification blares on the side indicating high stress levels throughout the night, tense muscles around the head and forecasting a feeling of headache that may prop up in the day, suggesting a dose of painkiller before work. The weather outside is reported as very good with no rains and work schedule is displayed after that.

The living room and kitchen is spick and span; the bath water exactly 37 degrees Celsius, the day’s clothes laid out- creaseless and ironed. Breakfast table is laid with 2 eggs, sunny side up.

The self-driving ride is on time with the first meeting on the way to work. The planned day runs smoothly. The bank account at 15:00 hours shows that the amount has increased as expected. The watch detects an exercise pattern in the evening, a slow walk for 60 minutes with increase in heart rate to warm up level. The 10,000 steps goal for the day was achieved.

Bright red – the notification remains visible on the side and beams again indicating nocturnal trend of high stress in the evening. A reminder to watch the trend is added to her digital notes.

**30 June 2051**

**06:00**

**Figure 1:**
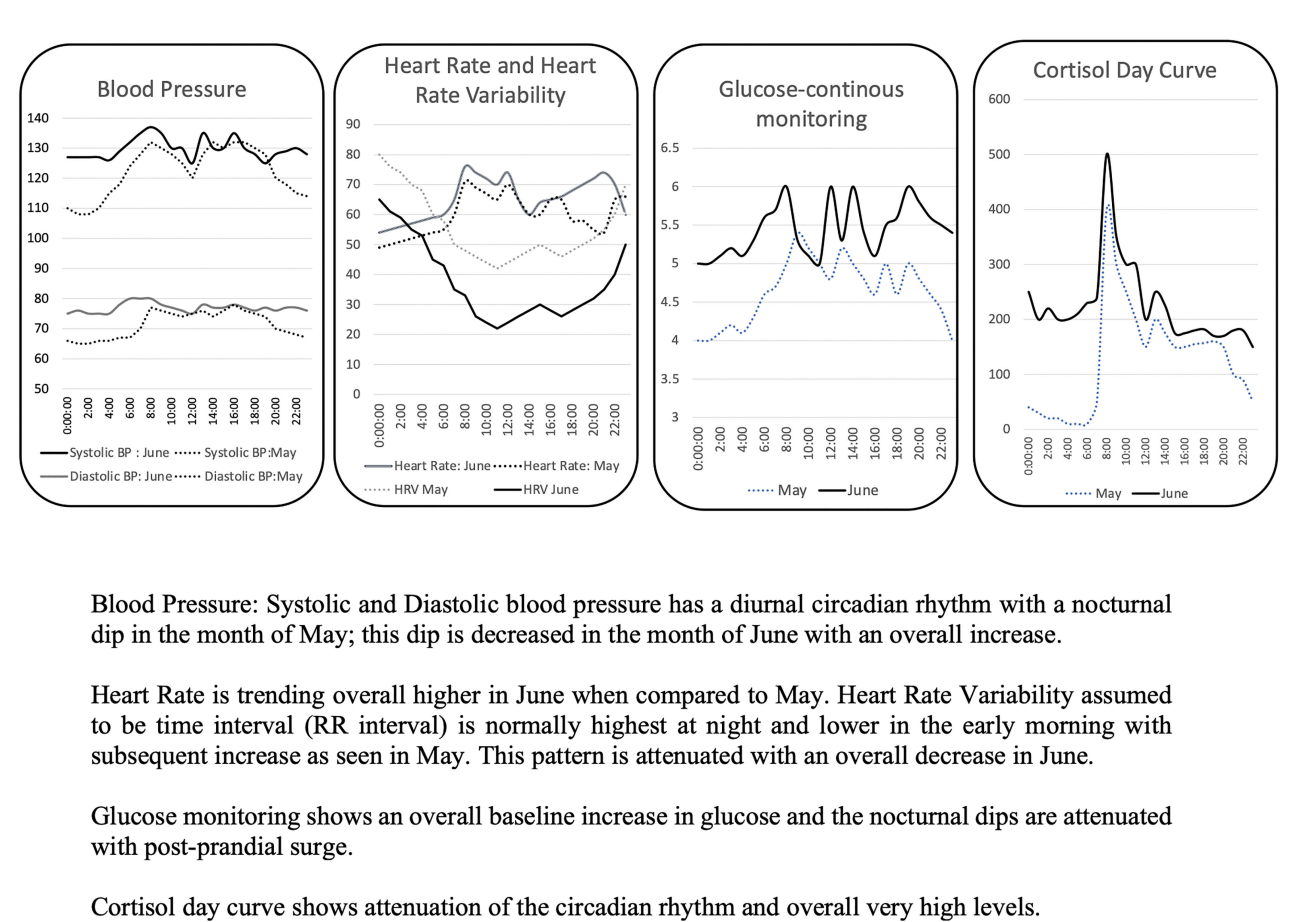
Deep Analytics interface showing the difference in daily patterns. Blood Pressure: Systolic and Diastolic blood pressure has a diurnal circadian rhythm with a nocturnal dip in the month of May; this dip is decreased in the month of June with an overall increase. Heart Rate is trending overall higher in June when compared to May. Heart Rate Variability assumed to be time interval (RR interval) is normally highest at night and lower in the early morning with subsequent increase as seen in May. This pattern is attenuated with an overall decrease in June. Glucose monitoring shows an overall baseline increase in glucose and the nocturnal dips are attenuated with post-prandial surge. Cortisol day curve shows attenuation of the circadian rhythm and overall very high levels.

Alarm beeps: Reminder: check the detailed analytics (**Figure 1**).

Heart Rate: Trending higher

Heart Rate Variability: Trending Lower

More information: see detailed analytics

Systolic Blood Pressure: Abnormal

More information: see detailed analytics

Diastolic Blood Pressure: Abnormal

More information: see detailed analytics

Glucose: Abnormal

More information: Baseline trend higher; see detailed analytics

Hypnograph: Awake.

Stress level: “Very High”

Cortisol Level: “High” from 00:00 to 24:00

More information: No Dip of cortisol levels at night; see detailed analytics

**1 July 2051**

**06:00**

Alarm beeps: **The stress hormones are in dangerous high levels, please visit the nearest healthcare facility for deeper analysis**.

The morning ride slows at the entrance of a healthcare facility and signals -disembarkation. Claire alights and enters the reception grumbling about the ‘obvious glitch in the watch’. They take the wearable and download all the data to the nearest workstation. Indeed, very high cortisol levels with a loss of the usual circadian rhythm in all parameters is observed. A higher order specialist workstation (termed Endocrinology) is assigned to Claire. She waits for her turn; foot tapping impatiently and enters the room. An endocrinologist is seated at the computer table. She smiles serenely and asks about the day, general feelings, and emotions. The Heart rate monitor reflects an increase, as Claire feels visibly uncomfortable sharing deep thoughts with a stranger. A new watch is given to her with an additional chip which would measure the levels at increased intervals, more precisely, throughout the day for next week and upload results to her doctor’s database. The situation would be reviewed in one week to decide upon the next course of action.

The week passes routinely. The only reminder is on the day of the appointment when the ride slows again in front of the healthcare facility. This time, the lady is whisked directly into her endocrinologist’s room. The data, uploaded live and pathways studied, shows high hormones -steroids with an active pituitary to adrenal pathway suggesting a pathology in the pituitary gland. The proposed diagnosis is explained, and a small chip is inserted underneath her collar bone. After the chip insertion, she is made to pass through a scanner and remain there for 5 minutes.

The screen confirms the diagnosis: Cushing’s disease: microscopic hot spot in pituitary. The next screen recommends an available specific targeted treatment to target this region deliverable through a small nano-based therapeutic implant on the forearm.

Claire is free to go after that. Daily circadian patterns, monitored through the watch, slowly returns to normal within a month. Every time a similar increase in cortisol levels over a threshold is seen, an additional dose is delivered remotely through the same implant.

No further alarms are heard as life continues as usual.

## Discussion

In the 20th century, practice of medicine and healthcare benefited from significant scientific breakthroughs. We are at inflection point for another incredible breakthrough in healthcare – in the sense that digitization will enable the application of data technologies and artificial intelligence into healthcare. The term ‘digital biomarker’ has been introduced. FDA defines a digital biomarker as “characteristic or set of characteristics, collected from digital health technologies, that is measured as an indicator of normal biological processes, pathogenic processes, or responses to an exposure or intervention, including therapeutic interventions” (1). This ability to derive biomarkers from daily patterns can potentially provide context to enrich normal values for the population, derive individual person-centric baseline values, and assess changes in health status over time to make clinical diagnosis. Modern-day wearables can be in the form of headbands, sociometric badges, camera clips, smart watches or sensors embedded in clothing and have the ability to monitor vital physiological measurements such as heart rate, electrocardiogram, heart rate variability, respiratory rate, oxygen saturation, temperature, pressure sensors, activity levels, sleep patterns, environmental sound and light exposure etc (2). The requirements for authorisation by the U.S. FDA or regulatory CE-marking, remuneration, and privacy/data security depend on the specificities of the product, its purpose, the technology, the risks and benefits, and the data it processes.

Devices with capability to measure blood pressure, in the form of multi-parameter, miniaturized solutions for home environments are currently being pursued with great interest (3). Correlations of ambulatory blood pressure, especially high nocturnal blood pressure with cardiovascular risk has been observed and automated methods of blood pressure monitoring are being encouraged (4). Various techniques are being exploited for these measurements including miniaturization of cuff oscillometry, tonometry, pulse propagation techniques and pulse wave analysis (3, 5). Pulse propagation techniques include using the PTT (pulse transit time) or the PAT (pulse arrival time) (time required for the pulse to travel between 2 arterial sites) is directly proportional to the blood pressure. Photoplethysmography (PPG) uses optical and inertial sensors to detect blood flow patterns. The technology indirectly measures the blood flow rate through the amount of light absorbed or reflected by blood vessels. Since the relationship between PPG and blood pressure is non-linear, a machine-learning algorithm is used to convert blood flow information to blood pressure measurement. As more data is collected, the algorithm will get more precise (3, 5). Protocols for validation of these ambulatory blood pressure measurements are being developed and some of these devices will likely receive regulatory approvals in the near future. Please see **Table 1** for a summary of devices with FDA approval or CE mark.

**Table 1:** Blood pressure monitoring devices with FDA approval or European CE Mark.

Device	Technology	Calibration	Regulatory Approval
1. Omron Heartguide (OMRON Corporation, Japan): wrist watch 2. Caretaker 4 (Caretaker Medical, US): wrist mounted with inflatable finger cuff	Cuff Oscillometric Method: Integration of miniature cuff into a smart watch	Self-calibration	1. FDA approval 2. FDA approval
3. BPro (Healthstats, Singapore)	Wrist watch radial artery -Tonometry	Requires calibration	FDA Approval
4. Biobeat	Pulse Arrival Time (PAT)	Requires calibration	FDA Approval
5. Aktiia	Photoplethysmography (PPG) and Pulse Wave Analysis	Requires calibration	CE Mark

Currently, non-invasive methods for glucose measurements in a simple wearable like a watch, are under development. Methods using a subcutaneous wired enzyme glucose sensor inserted in the body which transmits data to a smart phone are available and approved by the FDA (6–8) (**Table 2**). These systems can be applied by self (6, 7) or need to be implanted by a healthcare professional.

**Table 2:** FDA approved methods of non-invasive continuous glucose monitoring.

	Method	Frequency	Application	Duration	Calibration
1. Abbot Freestyle Libre systems	Subcutaneous wired enzyme glucose sensing technology	1 minute	Self	14 days	Factory-calibrated
2. Dexcom G6 system	Subcutaneous wired enzyme glucose sensing technology	5 minutes	Self	10 days	Factory-calibrated
3. Ever sense CGM systems	Fluorescent sensor	5 minutes	Healthcare provider	90-180 days	User calibrate 1-2 times a day

Cortisol rises early in morning and is highest before awakening, it falls naturally throughout the day and can spike in response to meals and to stress. Current methods for measuring cortisol concentrations is a laboratory-based blood test and is time consuming. Increasingly more rapid and direct plasma assays are being developed (9, 10). Salivary and sweat cortisol concentrations reflect the systemic steroid concentrations (11, 12). In terms of development, several independent researchers across the globe are working on systems which can be used to measure cortisol concentrations in body fluids and can thus be estimated on a superficial patch or wearable. A cortisol sensor has been formulated using extended gate-field-effect transistor (13–17). This has been developed as wearable contact lenses which can detect cortisol concentration in tears (13). This cortisol sensor is integrated with transparent antennas and wireless communication circuits to link with the smartphone (14). A similar sensing system applied on the wrist with capability to measure sweat cortisol levels has been developed and tested which shows promise (14–17) (**Table 3**). It is very likely that such a device will be developed and integrated into the traditional wearable watch as cortisol levels have applications in measurement of daily stress or allostatic load.

**Table 3:** Current and upcoming methods of cortisol assessment.

Test Principle	Sample	Time
1.EIA (competitive, chemiluminescence)	Serum/Plasma	18-40 min
2.ECLIA (Competitive electrochemiluminescence immunoassay)	Serum/Plasma/urine	18-40 min
3.CMIA (Competitive Chimiluminescence Microparticle Immunoassay)	Serum/Plasma/saliva	30 min
4.EIA (competitive, dry technology chemiluminesence)	Serum/Plasma/saliva/urine	10 min
5.LC-MS/MS	Serum/Plasma/saliva/urine	Varies depending on lab; direct measurement shortens time
6. Wireless immunosensing of cortisol through contact lenses	Tears	Instant
7. Graphene based wireless Wearable device	Sweat	Instant mobile technology

With regards the percentage of population using a wearable, whether the utopian type of order written in this short story can be true, is also highly probable. Insurance companies or other healthcare payers are likely going to mandate wearing of a daily wearable, so to enable preventive care. It is likely that the premium rates may be higher in individuals refusing to comply in the beginning but in the long run when the population adopts this technology, it will become a mainstay.

This lady above has ACTH-dependent Cushing’s syndrome secondary to a pituitary adenoma also called Cushing’s Disease (CD). Cushing’s disease was first described in a landmark monograph more than a century ago, in 1910 by Dr. Harvey Cushing. He described his first patient, Minnie G. to have “… syndrome of painful obesity, hypertrichosis, and amenorrhea, with overdevelopment of secondary sexual characteristics accompanying a low grade of hydrocephalus and increased cerebral tension. Pituitary, adrenal, pineal or ovary?” (18–21).

Diagnosis and management of CD has evolved significantly in the last century. Despite the advances, significant pitfalls and challenges remain. The typical patient presents 5-10 years into the illness, when the high cortisol hormones lead to downstream multi-organ problems. They present to healthcare when frank symptoms and signs are visible which includes significant change in appearance (moon shaped facies, central obesity) and change in metabolic status (hypertension, diabetes mellitus) and body composition (central visceral obesity and osteoporosis). After clinical suspicion, multiple tests (1mg dexamethasone suppression test, 24 hrs urine free cortisol, midnight salivary cortisol, ACTH, cortisol assays) are required to confirm the diagnosis. Once diagnosis is confirmed, then localisation is extremely challenging and pituitary adenomas secondary to Cushings’ is detected on magnetic resonance imaging with sensitivity ranging from 42% to 85%. Early, small lesions <4 mm in size are even more difficult to localise. Functional imaging, in the form of 11c-methionine PET, is still under research development. The invasive inferior petrosal sinus sampling needs to be performed which can localise the lesion at best to the pituitary gland only (21). Many of the tumours are sent for surgery without localisation and are localised intraoperatively (22). Surgical treatment is the mainstay for pituitary adenomas but remains challenging and only a handful of patients go into remission (at best 60-70%) (23). Medical treatment has evolved with 2 FDA approved therapeutics (pasireotide and mifepristone). However, even these are not superior to curative excision (24).

Early diagnosis in CD can be made through changes in heart rate and blood pressure dynamics (25, 26). The hypothalamic-pituitary-adrenal axis (HPA), responsible for the circadian rhythm of endogenous cortisol secretion, contributes to the circadian rhythm of blood pressure (26). In CD, the typical dip in nocturnal blood pressure (lower by 10% from baseline) is absent and the daytime heart rate is higher (25). Heart rate variability shows a characteristic pattern in terms of circadian differences and the typical pattern of highest between 10-2 PM at night is attenuated in Cushings disease (27). Corticosteroids also affects insulin signalling pathways directly and through an increase in growth hormone and results in higher post prandial glucose and blunted circadian pattern (28). A characteristic pattern has also been reported in patients with acromegaly, a pituitary condition with high growth hormones even before it affects glucose tolerance (29).

Differential diagnosis includes: Phaeochromocytoma, and primary aldosteronism. Periodic patterns would suggest phaeochromocytoma and similar pattern as CD with normal steroid concentrations suggest primary aldosteronism.

Adrenal and pituitary incidentalomas are commonly detected during screening for non- related medical concerns. These may represent subclinical hypercortisolism (30, 31) in otherwise clinically asymptomatic patients. The wearables can potentially be used to ascertain subclinical disease and to differentiate from pseudo-Cushings’ syndrome (occurs in obesity, alcoholism etc). Conversely, with the advent of regular wearables incorporating cortisol, it is possible that such subclinical glucocorticoid excess will be detected more frequently and may even be the causative mechanism in some patients with metabolic abnormalities. We envision that initially, the wearables will be useful in patients with clinical suspicion like above. However, with time as more long term longitudinal data is collected in the population (over 10-20 years), big data analytics is set to uncover digital biomarkers (patterns) that can be used to make an early diagnosis before definite clinical signs appear. We envision that by 2051, preventive care with remote digital monitoring is highly probable at a population scale level.

Next steps for localisation require the characterisation of CRH-ACTH-cortisol pathway (32). Cushings disease has a unique metabolomic signature (33) and with advancement in omics platforms (34), and in artificial intelligence predictive analytics it is highly probable that the pathway can be used to identify the active areas.

Pituitary lesions in Cushing’s syndrome are only detected by MRI in <60% of cases. Hybrid imaging combining PET and MRI such as 11C-methionine PET co-registered with volumetric MRI will likely improve the sensitivity and specificity in the near future (35). As novel data reveals more information on exact gene and protein expressions in these tumours, it will become possible to design advanced functional imaging methods which targets these areas to show “hotspots”.

Molecular targeted therapies such as ACTH antagonists (36) or melanocortin type 2 receptor (MC_2_R) (37), EGFR, retinoic acid receptors, CDK with specific inhibitors for CD, and cyclin E-Mediated Human Proopiomelanocortin pathway (38–42) are being developed. Efficiency in targeted delivery can be achieved with the conjugation of drugs with target cell surface-targeting moieties and encapsulation of unique nanocarriers/nanoparticles (43). Studies evaluating the clinical efficacy of these therapeutics will bring some of these into clinical practice.

While the above case vignette, appears to be a sci-fi fantasy and significant challenges in each area of diagnostics and therapeutics remain; the wearables and the massive data that will be accrued, will likely transform healthcare through predictive modelling and implementation of personalised care. One of the key factors for successful implementation is defining specific problems for targeted wearable solutions in specific disease states and establishing partnerships with clinician champions (44). We envision that these methods are set to bring about a major paradigm shift in the management of most endocrine related conditions. The practice of endocrinology is set to evolve significantly in the coming decades.

At the turn of the 20th century, Dr. William Osler said:

“Listen to your patient; he is telling you the diagnosis,”

In the 21st century:

“Look and analyse the digital physiological and behavioural trends; therein lies the diagnosis”.

## Data Availability Statement

The original contributions presented in the study are included in the article/supplementary material. Further inquiries can be directed to the corresponding author.

## Author Contributions

RD and BB conceptualized the short story, performed literature review, critically reviewed and wrote the final draft. SB reviewed and critically evaluated the final draft. All authors reviewed the final manuscript.

## Conflict of Interest

The authors declare that the research was conducted in the absence of any commercial or financial relationships that could be construed as a potential conflict of interest.

## Publisher’s Note

All claims expressed in this article are solely those of the authors and do not necessarily represent those of their affiliated organizations, or those of the publisher, the editors and the reviewers. Any product that may be evaluated in this article, or claim that may be made by its manufacturer, is not guaranteed or endorsed by the publisher.

## References

[1] Vasudevan S Saha A Tarver ME Patel B. Digital Biomarkers: Convergence of Digital Health Technologies and Biomarkers. NPJ Digit Med (2022) 5(1):36. doi: 10.1038/s41746-022-00583-z 35338234 PMC8956713

[2] Vijayan V Connolly JP Condell J McKelvey N Gardiner P. Review of Wearable Devices and Data Collection Considerations for Connected Health. Sensors (Basel) (2021) 21(16):5589. doi: 10.3390/s21165589 34451032 PMC8402237

[3] Panula T Sirkia JP Wong D Kaisti M. Advances in non-Invasive Blood Pressure Measurement Techniques. IEEE Rev BioMed Eng (2022). doi: 10.1109/RBME.2022.3141877 . Epub ahead of print 35015647

[4] Muntner P Shimbo D Carey RM Charleston JB Gaillard T Misra S. Measurement of Blood Pressure in Humans: A Scientific Statement From the American Heart Association. Hypertension (2019) 73(5):e35–66. doi: 10.1161/HYP.0000000000000087 PMC11409525 30827125

[5] Nachman D Gepner Y Goldstein N Kabakov E Ishay AB Littman R. Comparing Blood Pressure Measurements Between a Photoplethysmography-Based and a Standard Cuff-Based Manometry Device. Sci Rep (2020) 10(1):1–9. doi: 10.1038/s41598-020-73172-3 32999400 PMC7527983

[6] Blum A. Freestyle Libre Glucose Monitoring System. Clin Diabetes (2018) 36(2):203–4. doi: 10.2337/cd17-0130 PMC5898159 29686463

[7] Wadwa RP Laffel LM Shah VN Garg SK. Accuracy of a Factory-Calibrated, Real-Time Continuous Glucose Monitoring System During 10 Days of Use in Youth and Adults With Diabetes. Diabetes Technol Ther (2018) 20(6):395–402. doi: 10.1089/dia.2018.0150 29901421 PMC6110124

[8] Ascensia Diabetes Care Announces Fda Approval of the Eversense E3 Continuous Glucose Monitoring System for Use for Up to 6 Months. In: Ascensia Diabetes Care. Available at: https://www.prnewswire.com/news-releases/ascensia-diabetes-care-announces-fda-approval-of-the-eversense-e3-continuous-glucose-monitoring-system-for-use-for-up-to-6-months-301481042.html.

[9] J.Yeo KT Babic N Hannoush ZC Weiss RE. Endocrine Testing Protocols: Hypothalamic Pituitary Adrenal Axis. In: Feingold KR Anawalt B Boyce A, editors. TABLE I, Methods Available for Measurement of Serum or Plasma Cortisol. Available at: https://www.ncbi.nlm.nih.gov/books/NBK278940/table/endocrin-test-hpaa.tableimeth/.

[10] Ohlson S Kaur J Raida M Niss U Bengala T Drum CL. Direct Analysis - No Sample Preparation - of Bioavailable Cortisol in Human Plasma by Weak Affinity Chromatography (WAC). J Chromatogr B Analyt Technol BioMed Life Sci (2017) 1061-1062:438–44. doi: 10.1016/j.jchromb.2017.07.035 28820982

[11] Russell E Koren G Rieder M Van Uum SH. The Detection of Cortisol in Human Sweat: Implications for Measurement of Cortisol in Hair. Ther Drug Monit (2014) 36(1):30–4. doi: 10.1097/FTD.0b013e31829daa0a 24216536

[12] Cvijetic S Keser I Jurasović J Orct T Babić Ž Boschiero D. Diurnal Salivary Cortisol in Relation to Body Composition and Heart Rate Variability in Young Adults. Front Endocrinol (Lausanne) (2022) 13:831831. doi: 10.3389/fendo.2022.831831 35355570 PMC8959541

[13] Sheibani S Capua L Kamaei S Akbari SSA Zhang J Guerin H. Extended Gate Field-Effect-Transistor for Sensing Cortisol Stress Hormone. Commun Mater (2021) 2(1):10. doi: 10.1038/s43246-020-00114-x 33506228 PMC7815575

[14] Ku M Kim J Won JE Kang W Park YG Park J. Smart, Soft Contact Lens for Wireless Immunosensing of Cortisol. Sci Adv (2020) 6(28):eabb2891. doi: 10.1126/sciadv.abb2891 32923592 PMC7455488

[15] Torrente-Rodríguez RM Tu J Yang Y Min J Wang M Song Y. Investigation of Cortisol Dynamics in Human Sweat Using a Graphene-Based Wireless Mhealth System. Matter (2020) 2(4):921–37. doi: 10.1016/j.matt.2020.01.021 PMC7138219 32266329

[16] Cheng C Li X Xu G Lu Y Low SS Liu G. Battery-Free, Wireless, and Flexible Electrochemical Patch for *in Situ* Analysis of Sweat Cortisol *via* Near Field Communication. Biosens Bioelectron (2021) 172:112782. doi: 10.1016/j.bios.2020.112782 33157409

[17] Wang B Zhao C Wang Z Yang KA Cheng X Liu W. Wearable Aptamer-Field-Effect Transistor Sensing System for Noninvasive Cortisol Monitoring. Sci Adv (2022) 8(1):eabk0967. doi: 10.1126/sciadv.abk0967 34985954 PMC8730602

[18] Cushing H. The Basophil Adenomas of the Pituitary Body and Their Clinical Manifestations (Pituitary Basophilism). Bull Johns Hopkins Hosp (1932) 50:137–95. doi: 10.1002/j.1550-8528.1994.tb00097.x

[19] Cushing H. The Pituitary Body and its Disorders: Clinical States Produced by Disorders of the Hypophysis Cerebri. Philadelphia & London, JB Lippincott (1912).

[20] Lanzino G Maartens NF Laws ER Jr. Cushing's Case XLV: Minnie G. J Neurosurg (2002) 97:231–4. doi: 10.3171/jns.2002.97.1.0231 12134925

[21] Tabarin A Assié G Barat P Bonnet F Bonneville JF Borson-Charzot . Consensus Statement by the French Society of Endocrinology (SFE) and French Society of Pediatric Endocrinology & Diabetology (SFEDP) on Diagnosis of Cushing's Syndrome. Ann Endocrinol (Paris) (2022) 83(2):119–41. doi: 10.1016/j.ando.2022.02.001 35192845

[22] Sabahi M Shahbazi T Maroufi SF Vidal K Recinos PF Kshettry VR. MRI-Negative Cushing's Disease: A Review on Therapeutic Management. World Neurosurg (2022). doi: 10.1016/j.wneu.2022.03.076 35338018

[23] Zhang T Zhang B Yuan L Song Y Wang F. Superiority of Endoscopic Transsphenoidal Pituitary Surgery to Microscopic Transseptal Pituitary Surgery for Treatment of Cushing's Disease. Rev Assoc Med Bras (1992) (2021) 67(11):1687–91. doi: 10.1590/1806-9282.20210732 34909899

[24] Lau D Rutledge C Aghi MK. Cushing's Disease: Current Medical Therapies and Molecular Insights Guiding Future Therapies. Neurosurg Focus (2015) 38(2):E11. doi: 10.3171/2014.10.FOCUS14700 25639313

[25] Pecori Giraldi F Toja PM De Martin M Maronati A Scacchi M Omboni S. Circadian Blood Pressure Profile in Patients With Active Cushing's Disease and After Long-Term Cure. Horm Metab Res (2007) 39(12):908–14. doi: 10.1055/s-2007-992813 18046661

[26] Isidori AM Graziadio C Paragliola RM Cozzolino A Ambrogio AG Colao A. The Hypertension of Cushing's Syndrome: Controversies in the Pathophysiology and Focus on Cardiovascular Complications. J Hypertens (2015) 33(1):44–60. doi: 10.1097/HJH.0000000000000415 25415766 PMC4342316

[27] Chandran DS Ali N Jaryal AK Jyotsna VP Deepak KK. Decreased Autonomic Modulation of Heart Rate and Altered Cardiac Sympathovagal Balance in Patients With Cushing's Syndrome: Role of Endogenous Hypercortisolism. Neuroendocrinology (2013) 97(4):309–17. doi: 10.1159/000345905 23327928

[28] Sun Q Li X Chen P Chen L Zhao X. The Beta-Cell Function and Glucose Profile of Newly Diagnosed Acromegalic Patients With Normal Glucose Tolerance. Int J Endocrinol (2021) 2021:3666692. doi: 10.1155/2021/3666692 34917145 PMC8670947

[29] Ferraù F Korbonits M. Metabolic Comorbidities in Cushing's Syndrome. Eur J Endocrinol (2015) 173(4):M133–57. doi: 10.1530/EJE-15-0354 26060052

[30] Di Dalmazi G Pasquali R Beuschlein F Reincke M. Subclinical Hypercortisolism: A State, a Syndrome, or a Disease? Eur J Endocrinol (2015) 173(4):M61–71. doi: 10.1530/EJE-15-0272 26282599

[31] Reincke M Nieke J Krestin GP Saeger W Allolio B Winkelmann W. Preclinical Cushing's Syndrome in Adrenal "Incidentalomas": Comparison With Adrenal Cushing's Syndrome. J Clin Endocrinol Metab (1992) 75(3):826–32. doi: 10.1210/jcem.75.3.1517373 1517373

[32] Majzoub JA. Corticotropin-Releasing Hormone Physiology. Eur J Endocrinol (2006) 155:S71–6. doi: 10.1530/eje.1.02247

[33] Vega-Beyhart A Iruarrizaga M Pané A García-Eguren G Giró O Boswell L. Endogenous Cortisol Excess Confers a Unique Lipid Signature and Metabolic Network. J Mol Med (Berl) (2021) 99(8):1085–99. doi: 10.1007/s00109-021-02076-0 33881561

[34] Pînzariu O Georgescu B Georgescu CE. Metabolomics-A Promising Approach to Pituitary Adenomas. Front Endocrinol (Lausanne) (2019) 9:814. doi: 10.3389/fendo.2018.00814 30705668 PMC6345099

[35] Bonneville JF Potorac I Petrossians P Tshibanda L Beckers A. Pituitary MRI in Cushing's Disease - an Update. J Neuroendocrinol (2022) 15:e13123. doi: 10.1111/jne.13123 35352410

[36] Pivonello R Fleseriu M Newell-Price J Bertagna X Findling J Shimatsu A. LINC 3 Investigators. Efficacy and Safety of Osilodrostat in Patients With Cushing's Disease (LINC 3): A Multicentre Phase III Study With a Double-Blind, Randomised Withdrawal Phase. Lancet Diabetes Endocrinol (2020) 8(9):748–61. doi: 10.1016/S2213-8587(20)30240-0 32730798

[37] Goldenberg AJ Gehrand AL Waples E Jablonski M Hoeynck B Raff H. Effect of a Melanocortin Type 2 Receptor (MC2R) Antagonist on the Corticosterone Response to Hypoxia and ACTH Stimulation in the Neonatal Rat. Am J Physiol Regul Integr Comp Physiol (2018) 315(1):R128–33. doi: 10.1152/ajpregu.00009.2018 PMC6087887 29718699

[38] Fukuoka H Cooper O Ben-Shlomo A Mamelak A Ren SG Bruyette D. EGFR as a Therapeutic Target for Human, Canine, and Mouse ACTH-Secreting Pituitary Adenomas. J Clin Invest (2011) 121:4712–21. doi: 10.1172/JCI60417 PMC3226010 22105169

[39] Labeur M Paez-Pereda M Arzt E Stalla GK. Potential of Retinoic Acid Derivatives for the Treatment of Corticotroph Pituitary Adenomas. Rev Endocr Metab Disord (2009) 10:103–9. doi: 10.1007/s11154-008-9080-6 18604646

[40] Liu NA Jiang H Ben-Shlomo A Wawrowsky K Fan XM Lin S. Targeting Zebrafish and Murine Pituitary Corticotroph Tumors With a Cyclin-Dependent Kinase (CDK) Inhibitor. Proc Natl Acad Sci U.S.A. (2011) 108:8414–9. doi: 10.1073/pnas.1018091108 PMC3100964 21536883

[41] Liu NA Araki T Cuevas-Ramos D Hong J Ben-Shlomo A Tone Y. Cyclin E-Mediated Human Proopiomelanocortin Regulation as a Therapeutic Target for Cushing Disease. J Clin Endocrinol Metab (2015) 100(7):2557–64. doi: 10.1210/jc.2015-1606 PMC5393529 25942479

[42] Theodoropoulou M Reincke M. Tumor-Directed Therapeutic Targets in Cushing Disease. J Clin Endocrinol Metab (2019) 104(3):925–33. doi: 10.1210/jc.2018-02080 30535260

[43] Ezhilarasan D Lakshmi T Mallineni SK. Nano-Based Targeted Drug Delivery for Lung Cancer: Therapeutic Avenues and Challenges. Nanomed (Lond) (2022). doi: 10.2217/nnm-2021-0364 35311343

[44] Smuck M Odonkor CA Wilt JK Schmidt N Swiernik MA. The Emerging Clinical Role of Wearables: Factors for Successful Implementation in Healthcare. NPJ Digit Med (2021) 4(1):45. doi: 10.1038/s41746-021-00418-3 33692479 PMC7946921

